# Editorial: Genes, diseases, immunity and immunogenomics

**DOI:** 10.3389/fgene.2023.1218084

**Published:** 2023-06-06

**Authors:** Hifzur R. Siddique

**Affiliations:** Faculty of Life Sciences, Aligarh Muslim University, Aligarh, India

**Keywords:** genes, diseases, immunity, lncRNA, cell death

Genes transcribe both coding and non-coding RNA and are involved in regulating several biological processes, such as cell division, differentiation, cell death, and multiple signaling pathways (Statello et al., 2021). Non-coding RNAs (ncRNAs) modulate expression patterns of various genes, which play important roles in different diseases. Several studies have unraveled associations between aberrant ncRNA expressions and pathologies of human diseases (Singh et al., 2020; Yeh et al., 2023). Like other ncRNAs, recently discovered ncRNAs, circular RNAs (circRNAs) have also been found to play an important role in gene regulation via interaction with other biomolecules like nucleic acids, proteins, and microRNAs (miRNAs). In this context, they are good candidates for diagnosing multiple human diseases, including cancers, neurological diseases, and inflammatory diseases (Singh et al., 2022). In recent decades, several discoveries have been made with genome-wide or candidate gene approaches that have revealed significant insights into ncRNAs and immune interactions in different diseases. As a result, there is a growing interest in this field to know more about interconnected relationship between ncRNAs and immune environment (Yosef and Regev, 2016; Houck et al., 2018; Furman et al., 2019). On the other side, specific human coding gene variants that contribute to enhanced susceptibility or resistance against several diseases have been identified. These genes also profoundly affect other gene expressions in disease onset or/and progression (Choi et al., 2020; Fatma and Siddique, 2021; Fatma et al., 2022). Transforming growth factor beta-1 (TGFβ1) plays an important role in proliferation and differentiation of benign prostatic hyperplasia (BPH) stroma, but key downstream genes of TGFβ1 is yet to be explored more. Xiang et al. reported upregulations of TGFβ1 in BPH stroma compared to normal prostate stroma. They reported a total of 497 differentially expressed genes in primary prostatic stromal cells (PrSCs) with and without TGFβ1 stimulation. Their study reported some new insights into the role of TGFβ1 in BPH stroma and provided clues for identifying potential downstream mechanisms and targets. Feng et al. reported the expression profiles of whole coding and non-coding RNA transcriptomes for chronic obstructive pulmonary disease (COPD). They constructed lncRNA/circRNA -miRNA-mRNA ceRNA networks which may regulate TNF-α/NF-κB, IL6/JAK/STAT3 signaling pathways. This paper is important for further research on mechanism of post-transcriptional regulation of COPD, identifying novel targets for diagnosis and prognosis. Lastly, epigenetic changes in DNA and RNA are crucial in multiple diseases. Histone modifications or N1-methyladenosine methylation (m1A)-an important RNA methylation modification, regulates the development of many tumors.

LncRNAs remodel tumor immune microenvironment (TIME) by regulating the functions of tumor-infiltrating immune cells. It remains uncertain how TIME-related lncRNAs (TRLs) influence different immunotherapy in different cancers (Park et al., 2022). Khan et al. summarized the role of various ncRNAs on microcirculation, invasiveness, altered metabolism, microenvironment, and modulation of immunological environment. Hu et al. reported the signature of lncRNAs which influence TIME by regulating the functions of tumor-infiltrating immune cells in colorectal cancer (CRC). They reported how TRLs affect prognosis and immunotherapy response of CRC and are helpful in prognosis and immunotherapy response predictions. Wang et al. reported a lncRNA (DUXAP8) that has been shown to function as an oncogene in various human cancers. Their study revealed that DUXAP8 might serve as a prognostic biomarker and potential therapeutic target for different cancers.

Different types of cell death play a crucial role in various diseases (Li et al., 2020; Xie et al., 2023). Zhang et al. reviewed association between autophagy and acute pancreatitis. They highlight regulatory function of different genes in progression or suppression of diseases and target these genes as a potential therapeutic approach for disease management. Zhang et al. reported prognostic and tumor microenvironment characteristics of cuproptosis in bladder cancer by genomic analysis and might be helpful to personalized medicine. Yang et al. reported eight cuprotosis-related lncRNAs signature of head and neck squamous cell carcinoma (HNSCC) as prognostic predictors, which may be promising biomarkers for prognosis of HNSCC during immunotherapy. A recently identified programmed inflammatory cell death mode called pyroptosis plays a crucial role in different inflammatory diseases (Wu et al., 2022). Wang et al. reported a signature of pyroptosis-related lncRNAs (PRlncRNAs) in gastric cancer and their role in immunotherapy and chemotherapy. They identified 3 PRlncRNAs which may also be a potential therapeutic target in gastric cancer therapy. Another type of cell death, necroptosis, is a novel caspase-independent, programmed necrotic cell death distinct from other genetically controlled cell death types. Recent investigations reported that necroptosis is associated with multiple diseases’ pathogenesis, progression, and prognosis, including cancers (Rosenbaum et al., 2010; Khoury et al., 2020). However, molecular mechanisms have not been completely explored in different diseases. Zhang et al. identified necroptosis-related molecular subtypes directly linked to lung adenocarcinoma (LUAD) therapeutic response. They reported 67 necroptosis-related genes from 522 LUAD samples and reported importance of predicting overall survival and therapeutic benefits for LUAD patients. Wang et al. reported necroptosis-related lncRNAs, which are useful for predicting prognosis and immunotherapy of osteosarcoma. They also validated three 3 lncRNAs (AL391121.1, AL354919.2, and AP000851.2) which may be helpful in predicting the prognosis of overall survivability and guidance for immunotherapy.

Cardiomyopathy is a major concern nowadays, often leading to progressive heart failure and sudden cardiac death. Based on machine learning, Ye et al. reported molecular subgroups in dilated cardiomyopathy and identified novel biomarkers. Further, they observed that patients from different molecular subgroups have unique gene expression patterns and clinical characteristics. This study is an important addition to precision medicine. Xu et al. discussed the role of exosomal miRNAs in atherosclerosis, myocardial injury and infarction, heart failure, aortic dissection, myocardial fibrosis, ischemic reperfusion, atrial fibrillation, and other diseases. Further, they explained the characteristics and aspects of exosome separation, extraction, and identification. Intimal hyperplasia (IH) is a prominent pathological event during in-stent restenosis and atherosclerosis in coronary heart disease. Zhang et al. reported ferroptosis-related genes’ expression profiles and functions in IH induced by carotid artery ligation in mice. Thirty-one ferroptosis-related genes (FRGs) showing significantly different expression were identified from 1,556 differentially expressed genes (DEGs) 14 days after ligation. They reported DEGs related to ferroptosis and IH and provided more evidence about ferroptosis’s role in IH.

Several specific genes regulate immunomodulatory molecules, such as IL2, IL3, miR-34a, and miR-17-92 (Olive et al., 2013; Taheri et al., 2020; Sarsenova et al., 2022). Besides, some molecules regulate immune responses by interacting with molecules related to immune response either directly or via regulating other molecules. Thus, these genes connect immunomodulatory pathways and shift pro-inflammatory balance towards pro-disease condition. Peng et al. identified and validated neurotrophic factor-related genes (NFRGs) signature in HNSCC to predict survival and immune landscapes. Due to heterogeneous nature and complex tumor microenvironment, outcome of immunotherapeutic of HNSCC patients is not so successful. They reported that 18 NFRGs are closely associated with HNSCC prognosis and could be good predictors of HNSCC. A nomogram based on this model can help clinicians classify HNSCC patients prognostically and identify specific subgroups of patients who may have better outcomes with immunotherapy and chemotherapy, and helpful for personalized treatment for HNSCC patients. Role of anoikis in clear cell renal cell carcinoma (ccRCC) remains unclear. Chen et al. reported a prognostic signature associated with immune infiltration landscape in ccRCC. They integrated multiple anoikis-related genes to establish a risk-predictive model which might be helpful for personalized treatment of ccRCC patients. Lin et al. investigated whether circulating NAD^+^ metabolism-related genes could be used to predict immunotherapy response in ovarian cancer (OC) patients. They found three different subgroups based on NMRGs expression patterns. Their prognostic signature has potential predictive value for OC prognosis and immunotherapy response.

Research on immunogenomics in different diseases has been gaining particular attention in recent decades. Recently, advances in immunogenetics have made reprogramming specificity and function of innate/adaptive immune cells possible, which leads to the promise of generating “pharmacological targets” that can respond to reprogrammed immune cells in disease conditions like inflammatory diseases or cancer. Calcific aortic valve disease (CAVD) has become a primary cause of aortic valve stenosis, insufficiency, and the most prevalent valvular heart disease. By meta-analysis, Wu et al. reported key immune-related genes (IRGs) and immune infiltration patterns in CAVD. A total of 220 differentially expressed IRGs were identified, and enrichment analysis of differentially expressed IRGs showed that they were significantly enriched in inflammatory responses. This meta-analysis suggested that PTPN11, GRB2, PTPN6, SYK, and SHC1 might be key differentially expressed IRGs associated with immune cell infiltration and might play a role in CAVD.

The prevalence of adult degenerative diseases is increasing at an alarming rate. However, molecular research related to these diseases is in an infant stage. Zhao et al. described the recently developed machine learning-based characterization of cuprotosis-related biomarkers and immune infiltration in Parkinson’s disease (PD). Three PD datasets from GEO database were combined after eliminating batch effects and identified 03 cuprotosis-related genes, ATP7A, SLC31A1, and DBT, associated with immune cells or immune function in PD and more accurate for diagnosis of PD course. The study reveals that several newly identified cuprotosis-related genes intervene in progression of PD through immune cell infiltration. Shi et al. investigated degenerative scoliosis (ADS)-associated mRNAs and lncRNAs by RNA-seq and performed comprehensive bioinformatics analysis based on lncRNA-mRNA co-expression network and protein-protein interaction (PPI) network. A total of 1,651 upregulated and 1,524 downregulated mRNAs and 147 upregulated and 83 downregulated lncRNAs were screened out from RNA-Seq data. This study provides insight into the altered transcriptome profile of long-stranded non-coding RNAs associated with ADS, which paves the way for further exploration of clinical biomarkers and molecular regulatory mechanisms for this poorly understood degenerative disease.

Wang et al. reported N1-methyladenosine methylation-related metabolic genes signature and subtypes for predicting prognosis and immune microenvironment in osteosarcoma. Also, to better guide individualized treatment, they analyzed immune checkpoint expression differences and drug sensitivity in different risk groups and clusters. They reported a prognostic signature, which may help to assess patient prognosis and immunotherapy response. Awal et al. reported a structural-guided identification of a small molecule inhibitor of ubiquitin-like containing plant homeodomain ring finger 1 (UHRF1) methyltransferase activity**-**a cell-cycle-regulated multidomain protein**.** Through molecular docking, they screened a dataset of 709 natural compounds where chicoric acid and nystose show higher binding affinities to the SRA domain. The study reported that chicoric acid could become a possible epidrug-like inhibitor against SRA domain of UHRF1 protein.

In conclusion, this Research Topic is a Research Topic of informative research articles, excellent reviews, and meta-analyses. I anticipate that this Research Topic contributes to expanding research community’s knowledge about this recent and rapidly growing field of genes, ncRNAs diseases, and immunity for a further thorough investigation, which will surely help to manage multiple deadly diseases.
